# Family Process and Peer Influences on Substance Use by Adolescents

**DOI:** 10.3390/ijerph10093868

**Published:** 2013-08-27

**Authors:** Alice Yuen Loke, Yim-wah Mak

**Affiliations:** School of Nursing, The Hong Kong Polytechnic University, Hong Kong, China; E-Mail: hsywmak@polyu.edu.hk

**Keywords:** family structure, family process, parental style, substance use, adolescents

## Abstract

This study explores the association of family process and peer influences with risk behaviors of adolescents. A total of 805 students were recruited from secondary schools. The results showed that adolescents who have parents who are “authoritarian” (OR = 1.856) were more likely to smoke. Adolescents who have conflicts with their parents (OR = 1.423) were more likely to drink. Those who have parents who are “permissive” were less likely to drink (OR = 0.885). Having friends who smoked (OR = 5.446) or drank (OR = 1.894), and friends’ invitation to smoke (OR = 10.455) or drink (OR = 11.825) were the dominant contributors to adolescent smoking and drinking. Interventions are needed that recognize the strength of the parent-child relationship, as well as strengthen family functioning through improved interpersonal, parenting, and monitoring skills.

## 1. Introduction

Young people are vulnerable of many behaviors that put their health at risk such as experimenting with smoking cigarettes, taking illegal drugs, and drinking alcohol [1]. A Hong Kong population-based survey reported that adolescents start to experiment with behaviors that are risky to their health when they are as young as 10 [2]. It was reported that among children aged 11–14 who had experimented with smoking cigarettes, 22.1% of them had smoked their first cigarette at the age of 10 or younger, and 60% had done so between the ages of 11–14. Among the 5% of children aged 11–14 who had drunk alcohol, about one-third had their first drink at the age of 10 or younger, and 44.3% between the ages of 11 and 14. Among children aged 11–14, 0.2% reported that they had taken illicit drugs in the 30 days preceding the survey.

The healthy development of adolescents assures a healthy society in the future. Behavior practiced by adolescents that puts their health at risk will extend into adulthood, rendering them vulnerable in adulthood to preventable morbidities and mortalities. The family process plays a key role in socializing and shaping children to enable them to adjust to the demands of their social environment [3]. Adolescent perceptions of what goes on in their family can make them vulnerable to behavior that is harmful to their health. Public health professionals are increasingly recognizing the major role that families play in a range of social and psychological problems affecting adolescent behaviors and health.

### 1.1. Family Process and the Impact on Adolescents

There is evidence showing that the family process plays a key role in inducing social and behavioral problems among youths or in protecting them from such problems. Family warmth and connectedness serve as a protective factor against many of the risky behaviors engaged in by adolescents [3]. Although childbirth comes naturally to most married couples, this is not the case with parenting skills. The family—in particular the parents—is responsible for setting an example in terms of health-promoting behaviors, and for providing protection against the uptake of behaviors harmful to health. Parents serve as role models for their children in imparting important health-related knowledge and appropriate behavior, as gatekeepers to both opportunities and barriers, and as the major source of reinforcement of behavior in most children [4,5].

The structure of the family affects the development of children. The children of divorced parents have been found to be twice as likely as children from intact families to display a lower level of conduct and delinquent behavior [6]. Adolescents who do not live with two parents are more likely to smoke cigarettes, and use marijuana and other illicit drugs. Studies have reported that adolescents from intact families (two birth parents) are less likely to be regular drinkers [7] and smokers [8] than those from either reconstituted or single parent families.

Studies have shown the relationship between the family process and behaviors by adolescents that put their health at risk. The family process has multiple characteristics, including: family connectedness, the satisfaction of family roles, and family conflicts. A study investigating the relationship between the influence of the family and behaviors among adolescents that put their health at risk reported that family connectedness was significantly inversely associated with alcohol and drug use [9]. The satisfaction of family roles, with positive mother-adolescent communication and quality father-adolescent communication, was associated with lower levels of problem behavior among adolescents [10]. Getting into conflicts with one’s parents also increases the likelihood that a young person will engage in risky health behavior. A cross-sectional study revealed that higher levels of parent-adolescent conflict (from the perspectives of both children and parents) are generally related to more behavioral problems among adolescents [11].

Parental warmth and support, and consistent and moderate discipline can inhibit behavioral problems in children [12]. Supportive parenting and positive perceptions of the quality of the parent-child relationship were also related to a reduction in behavioral problems among adolescents [13]. Parenting patterns are classified as: warm-directive, indulgent, authoritarian, and neglectful. Adolescents who perceived their parents as authoritative (high control, low support) and neglectful (low support and low control) were three times more likely than those who perceived their parents as warm-directive to report that they engaged in moderate or heavy alcohol use, substance use (2.75 times), and smoking (25% *vs*. 16.6%) [14].

A study that explored parental monitoring and negotiated unsupervised time found that adolescents with parental monitoring when compared with those without monitoring from their parents were less likely to drink (OR = 0.63), smoke (OR = 0.99), and take illicit drugs (OR = 0.73) [15]. The adolescents who have more unsupervised time from their parents were more likely than those with less unsupervised time from their parents to drink (OR = 1.85), smoke (OR = 1.35), and take illicit drugs (OR = 1.77). Another study showed that young adolescents who characterized their parents as neglectful were more likely to have tried smoking (OR = 4.27), drinking (OR = 1.99), and illicit drugs (OR = 3.34) than adolescents with warm and directive (authoritative) parents [16]. Parents’ perception of their own parental style also revealed similar phenomenon. The study showed that parents’ own report of high level of support and monitoring of their adolescents were associated with adolescents’ less involvement in smoking and drug taking [17].

### 1.2. Peer Influence and the Risk Behavior of Adolescents

Adolescents have a moderate to strong influence impact on their peers’ risk behavior [18]. Adolescents are susceptible to peer influence in that it has been observed that they are more likely to engage in risk taking in groups than alone. Adolescents, with their limited degree of self-reliance, which interferes with their ability to act independently of the influence of their peers, may be more easily swayed towards engaging in risky behavior [19]. Impulse control or sensation seeking by adolescents also plays an important role in the degree to which they might engage in risk taking behavior [20]. The results of a study that examined risk taking and decision making supported the idea that adolescents are more inclined than those of other age groups to engage in risky behavior and make risky decisions, and that peer influence plays an important role in explaining such behavior during adolescence [21].

A study involving 1,969 adolescents showed that a friend’s cigarette smoking, alcohol drinking, and drug use activities, significantly predicts an adolescent’s risk activities over a one-year period [22]. Peer influence is an outcome of peer selection processes and socialization [23]. Adolescents tend to affiliate and develop friendships with peers who shares common attitudes and characteristics contributing to homogeneity of peer group [24,25,26]. A study has reported that school-aged children with smoking parents were less likely to consider that smoking is harmful to health (67.7%), and had more peers who smoked (30.3%) [27].

Peer influence is also one of the top reasons given by adolescents for why someone their age would pick up smoking. Adolescents do offer cigarettes to their peers and that smoking is typically initiated in the context of peer groups [28]. ‘Cigarette offer by peers’ was a common reason given by adolescents for their picking up of their first cigarette (43.3%) [24]. Adolescents also tend to pick up their cigarettes when they were ‘wanting acceptance from friends’ (36%) [29].

A study has reported that adolescents who have a smoking peer are four times more likely to smoke than those who do not [27]. It was concluded that peers may also play a crucial role in the development of adolescents by influencing how they interpret information on risk behaviors and shaping their normative beliefs. A study found that having a friend who discourages a teen from engaging in particular types of behavior may also protect adolescents from engaging in risky activities [22].

Public health professionals are increasingly recognizing the key role that families and peers play in a range of social and behavioral problems affecting young adolescents. To address youth problems in the community, such as substance use, preventative action must be taken—for example, looking into adolescent perceptions of the family process and the peers that surround them, and identifying the issues that put adolescents in a vulnerable position.

This study was conducted to examine the family process of adolescents and the influence of their peers, and how both factors relate to the issue of substance use by adolescents. A better understanding of the family process responsible for behaviors by adolescents that are harmful to their health will provide the information needed to design appropriate interventions for families that are aimed at supporting these families and responding to the needs of those with at-risk adolescents.

## 2. Methods

The aim of this study was to explore the influence of family structure, the family process, parenting style, and the influence of friends on substance use by adolescents. This is a cross-sectional survey that employed a self-developed questionnaire. The study was conducted in a district in Hong Kong populated mostly by families of a lower socio-economic class, where a high prevalence of risky behaviors among adolescents has been reported.

### 2.1. Subject Recruitment

Secondary school principals in the Sham Shui Po District were approached for permission to conduct the survey in their schools. Prior to the survey, adolescents of the participating schools were given an information sheet and a refusal form to bring home to their parents. The information sheet provided to the parents clearly stated the purposes of the survey, and those parents who did not want their children to complete the questionnaire were asked to return the refusal form.

Five secondary schools (including one all-boys school) in the district accepted the invitation to participate in the survey. Two classes (Forms 1–6) were randomly selected from each school. The participating students completed the survey in a regular classroom setting on an agreed-upon date and time that did not interfere with their school schedule. A member of the research team distributed and collected the questionnaires.

### 2.2. Questionnaires

The questionnaire consisted of questions on the students’ perception of the family process; substance use (smoking, drinking alcohol, and using drugs), their parents’ and friends’ smoking behavior and their acceptance of smoking, the demographic characteristics of the adolescents, and their family structure (if they live with both parents or with a single parent).

The family process was examined from different perspectives, including family structure, family process (family activities, family conflicts), parental style (the interaction of support and control), and satisfaction with the fulfillment of the roles played by one’s parents and oneself [30]. The question on family structure asked if an adolescent was living with both birth parents or was from a single parent family. Family process was measured by the scales for “family activities” (six items), “conflicts with parents” (five items) and “parental support and control” (six items) adopted and modified from Sweeting *et al*. [31] and Glendinning *et al*. [8], respectively. Family activities refer to time spent in shared family activities, which include spending time together to “watch television”, “play indoor games”, “eat dinner”, “exercise”, “shopping”, and “visit relatives”. Family conflicts refer to arguments over everyday matters, which include “school works”, “cleaning own room”, “how money is spent”, “help in house chores”, and “choice of friends”. These two aspects were measured in terms of frequency. The items related to parental support and control include “cannot get on well”, “expect too much of me”, and “disappointed in me” for parental support; and “strong view about my appearance”, “disapproval of some of my friends”, and “want to know where I go in the evenings” for parental control. The parental support and control items were rated on a five-point Likert scale from “0-strongly disagree” to “5-strongly agree”. The questions on satisfaction with the fulfillment of the roles of parents and self were statements asking the respondents to indicate their satisfaction with each parent and with themselves using a five-point Likert scale ranging from “never” to “almost always”.

The questions on the adolescents’ substance use behaviors: smoking, drinking alcohol, and using illicit drugs, were adopted from various studies [32,33,34]. With regard to smoking and drinking alcohol, the students were categorized into never/ever tried/occasional/regular smokers; and never/ever tried/and occasional drinkers. Items on the adolescents’ illegal drug use, the smoking behavior of their family members and peers, and their family members’ and peers’ acceptance of smoking, were rated in terms of yes and no responses.

The developed questionnaire was translated into Chinese, and the content validity was assessed by a panel of three experts. All of the experts agreed that the items were relevant, given a Content Validity Index of 1.

### 2.3. Ethical Considerations

Ethical approval was obtained from the Human Subjects Research Ethics Committee of the University prior to the study. Permission to conduct the survey was also obtained from the principals of the schools attended by the participants. An information sheet explaining the aims of the study and a refusal form were sent to the parents. Students who returned the refusal forms were excluded from the study; all of the others completed the questionnaire in class. No risk in participating in this study was anticipated. The participants were ensured of confidentiality and anonymity, and assured that all data collected would be used solely for research purposes.

### 2.4. Data Analysis

All of the data were analyzed using the Statistical Package for the Social Sciences (SPSS) version 17 (SPSS Inc., Chicago, IL, USA). Descriptive statistics such as frequencies and percentages were used for the demographic data. The dependent variables, smoking status and drinking status, were dichotomized into “non-smoker” and “smoker” (including students who had ever tried smoking, smoked occasionally, and regular smokers); and “non-drinker” and “drinkers” (including students who had tried drinking alcohol, drank occasionally, and regular drinkers). Chi-square tests were used to compare the adolescents who had and had not engaged in substance use (smoking and drinking) with reference to family structure, family process, parental style, satisfaction with the roles of parents and self, the smoking behavior of family members, and the influence of friends.

A composite score was computed for the overall scale and for each of the subscales for measuring family process (family activities time, conflicts with parents, parental support and control). T-tests were employed to examine the relationship between the characteristics of the family process and the substance use (smoking and drinking) of the adolescents.

The composite scores of the scales for parental support (mean = 7.89, SD = 2.48) and control (mean = 3.53, SD = 2.28) were further divided into three groups representing a relatively high, moderate and low level of family support and control as perceived by the adolescents. Parental support is desirable, with the three statements were negatively stated, the Likert scale scores for “never” was given a score of 4 and “seldom” was given a score of 3, and almost always a score of 0, *etc*. The higher the score, the higher level of parental support there was. One would expect that only those parents who “never or seldom” cannot get on well, expect too much or disappointed in adolescents to be considered supportive (score 9 or higher, reported of at least “seldom” doing so in all 3 behaviors. The parents who were considered “sometimes” (score of 2) or “often” (score of 1) showing disapproval all three statements were considered non-supportive (score 6 or less). The middle range would be those who are moderate of parental support (score 7–8).

While on the other hand, the three statements on parental control are undesirable. The higher the score, the higher level of parental control there was. The parents who were considered as “often” doing so (score of 3) in one and “sometimes” (score of 2) in other two behaviors, would be considered of highly control (score 7 or higher). The parents who score of 4 or less (at least 2 statements being considered as seldom doing so) would put parents considered of being less controlling. The middle range would be those who are moderate of parental control (score 5–6). The three support and control categories were regrouped to produce five distinct types of parenting styles, namely “neglectful” (low support and low control)”; “authoritarian” (low support but high control); permissive (high support but low control); “warm-directive” (high support and high control); and “moderate” (moderate support and control) [14].

The two subscales measuring “family activity time” (mean = 11.53, SD = 4.314) and “conflict with parents” (mean = 5.90, SD = 3.748) were re-coded into two groups: frequent activity time with family” (score ≥ 12) and “little activity time with family” (score ≤ 11); and “have conflicts with parents” (score ≥ 11) and “not in conflict with parents” (score ≤ 10) in the binary logistic regression analysis. Logistic regression was used to identify the factors contributing to the substance use of adolescents.

## 3. Results

A total of 835 questionnaires were collected in the five schools. Only a few students, 2–4 students from each school returned their parents’ signed refusal forms and did not complete the questionnaires. A total of 30 cases were excluded due to incompleteness; as a result, 805 cases were included in the analysis for this study.

Table 1 shows the demographic characteristics of the respondents. There were more boys than girls in this study (73.4% *vs*. 26.6%). A majority of the adolescents had never smoked (91.1%), 4.5% had tried smoking, 1.7% smoke occasionally, and 2.7% were regular smokers. More adolescents had drunk alcohol (25.3%), with 30.7% of them drinking occasionally. Less than 1% of adolescents (*n* = 8) reported ever having used illegal drugs. Nearly 80% of the adolescents lived with both parents and had siblings. Adolescents from single parent families comprised 17.4% of the respondents.

**Table 1:** Demographic Characteristics of the Adolescents in the Study (*n* = 805).

Demographic characteristics		N	%
***Gender***			
	Male	591	73.4
	Female	214	26.6
***Age groups***			
	11–15	394	48.9
	16–18	411	51.1
**Substance use status**			
***Smoking***			
	Never smoke	733	91.1
	Has tried smoking	36	4.5
	Smoke occasionally	14	1.7
	Regular smoker	22	2.7
***Drinking alcohol***			
	Never drink	330	40.9
	Has tried drinking	228	25.3
	Drink occasionally	247	30.7
***Illegal drug use***			
	Has tried illegal drugs	8	<1.0
***Family structure***			
	Lives with both parents	641	79.6
	Lives with a single parent	140	17.4
	Has siblings	635	78.9
	Lives with siblings (among those with siblings)	575	90.6
***Smoking status of***			
	Father	359	44.6
	Mother	45	5.6
	Sibling	63	7.8
	Friend(s)	169	22.9
***Smoking status of***			
	Father	359	44.6
	Mother	45	5.6
	Sibling	63	7.8
	Friend(s)	169	22.9
***Satisfaction with role fulfillment***			
	of father	413	51.7
	of mother	507	63.2
	of self as a child	361	45.1
***Parenting styles***			
	Moderate (moderate support and control)	350	43.2
	Permissive (high support and low control)	310	38.5
	Neglectful (low support and low control)	88	10.9
	Authoritarian (low support and high control)	52	6.5
	Warm-directive (high support and high control)	5	0.6

Nearly half of the respondents had a father who smoked (44.6%), while a respective 5.6% and 7.8% of the adolescents had a mother and sibling(s) who smoked. About a quarter (22.9%) of the adolescents had at least one friend who smoked. More adolescents were satisfied with their mother’s role fulfillment (51.7%) than with their father’s (43.2%) and their own role fulfillment as a child (45.1%).

The calculation of the composite scores for family support and control showed that most of the parents (43.2%) were moderate in their support and control of their children, 38.5% were permissive, 10.9% were “neglectful”, 6.5% were “authoritarian”, and only 0.6% were “warm and directive” in their style of parenting (Table 1).

Table 2 shows the comparison between adolescents who do and do not use substances, smoke, and drink, with reference to the smoking behaviors of family members, family structure, satisfaction with the roles of one’s parents and oneself, and the influence of friends. Those defined as smokers are those who had ever tried smoking or who smoke occasionally or regularly, and those defined as drinkers are those who had ever tried drinking. No comparisons were made for drug use since less than 1% of adolescents admitted to having tried illicit drugs.

The comparison between smoking and non-smoking adolescents showed that more smokers than non-smokers have a father who smokes (62.5% *vs*. 42.9%, *p* = 0.002), a mother who smokes (22.2% *vs*. 4.0%, *p* ≤ 0.001), and siblings who smoke (25.0% *vs*. 6.1%, *p* = 0.001). Fewer smokers than non-smokers were living with both parents (62.5% *vs*. 81.0%, *p* ≤ 0.001) and perceived that their family objected to their smoking (72.2% *vs*. 84.9%, *p* ≤ 0.003). The smokers were less likely than the non-smokers to be satisfied with the role fulfillment of their father (29.1% *vs*. 53.2%, *p* ≤ 0.001), mother (47.2% *vs*. 64.2%, *p* = 0.003), and their own self as a child (23.6% *vs*. 46.6%, *p* ≤ 0.001). More of the smokers than the non-smokers had friends who smoke (66.6% *vs*. 16.4%), and would accept a friend’s offer to smoke (48.6% *vs*. 4.0%), but had fewer friends who object to smoking (33.3% *vs*. 63.9%), all with *p* ≤ 0.001 (Table 2).

**Table 2:** Comparison of smoking behaviors of family members, family structure, satisfaction of roles, and friends’ factors between adolescents with and without risk behaviors (*n* = 805).

		Non-smoker		Daily Smoker			Chi-square Test			Non-drinker		Drinker		Chi-square Test	
		*n*	%	*n*	%					*n*	%	*n*	%		
	Total	733	91.1	72	8.9		*χ^2^*	*p*		330	40.9	475	59.0	*χ^2^*	*p*
**Smoking behavior of family members**															
	Father smokes (yes)	314	42.9	45	62.5	10.06			0.002 *	126	38.1	231	48.6	7.75	0.005 *
	Mother smokes (yes)	29	4.0	16	22.2	41.21			≤0.001 **	10	3.0	34	7.1	6.22	0.013 *
	Siblings smoke (yes)	45	6.1	18	25.0	29.77			≤0.001 **	10	3.0	53	11.2	17.85	≤0.001 **
**Family structure**															
	Lives with both parents	594	81.0	45	62.5	13.8		≤0.001 **		273	82.7	368	77.5	3.31	0.068
	Lives with a single parent	116	15.8	24	33.3	14.51		≤0.001 **		44	13.3	96	20.2	6.28	0.012 *
	Lives with siblings (yes)	532	72.6	56	77.8	0.74		0.389		241	73.0	344	72.4	0.27	0.603
****	**Family objects to smoking**	623	84.9	52	72.2	9.07		≤0.003 *		-	-	-	-	-	-
**Satisfaction with role fulfillment**															
	of father (yes)	390	53.2	21	29.1	15.1		≤0.001 **		179	54.2	232	48.8	2.66	0.103
	of mother (yes)	471	64.2	34	47.2	8.59		0.003 *		214	64.8	291	61.3	1.70	0.193
	of self as child (yes)	342	46.6	17	23.6	14.5		≤0.001 **		159	48.1	200	42.1	3.74	0.053
**Friends’ factors**															
	Have friends who smoke/drink	120	16.4	48	66.6	96.82		≤0.001 **		36	10.9	133	28.0	30.46	≤0.001 **
	Will accept friend’s invitation to smoke/drink	30	4.0	35	48.6	172.68		≤0.001 **		57	17.3	343	72.2	222.63	≤0.001 **
	Friend objects to smoking/drinking	469	63.9	24	33.3	31.85		≤0.001 **		211	64.9	283	59.0	5.47	0.019 *

* *p* ≤ 0.05, ** *p* ≤0.001.

The comparison between drinkers and non-drinking adolescents showed that more of the drinking than non-drinking and smoking adolescents have a father (48.6% *vs*. 38.1%, *p* = 0.005), mother (7.1% *vs*. 3.0%, *p* ≤ 0.013), and siblings (11.2% *vs*. 3.0%, *p* ≤ 0.001) who drink. More of the drinkers than non-drinkers were living with a single parent (20.2% *vs*. 13.3%, *p* = 0.012). Fewer drinkers than non-drinkers were satisfied with the role fulfillment of their father (48.8% *vs*. 54.2%), mother (61.3% *vs*. 64.8%), and their own self as a child (42.1% *vs*. 48.1%), but there were no statistically significant differences. More of the drinkers than non-drinkers had friends who drink (28.0% *vs*. 10.9%, *p* ≤ 0.001), and would accept a friend’s offer to drink (72.7% *vs*. 17.3%, *p* ≤ 0.001), but fewer had friends who object to drinking (59.0% *vs*. 64.9%, *p* = 0.019) (Table 2).

A comparison was made of the family process (family activities, conflicts with parents, and parental support and control) of adolescents who smoke/drink and those who do not (Table 3). The results show that when compared with non-smokers, smokers have a lower mean score for “time spent with parents” (mean = 9.06 *vs*. 11.78, *p* ≤ 0.001) and a higher mean score for “conflict with parents” (mean = 7.24 *vs*. 5.77, *p* = 0.002). The smokers, in comparison with those who do not smoke, also tended to think that their parents were providing them with less support (mean = 6.81 *vs*. 7.99, *p* ≤ 0.001) and exerting more control (mean = 4.32 *vs*. 3.45, *p* = 0.002). With regard to the comparison between drinkers and non-drinkers, the former have a higher mean score for “conflict with parents” (mean = 6.30 *vs*. 5.29, *p* ≤ 0.001). The drinkers, when compared with those who do not drink, also tended to think that their parents provide less support (mean = 7.58 *vs*. 8.33, *p* ≤ 0.001) and exert more control (mean = 3.83 *vs*. 3.09, *p* ≤ 0.001).

**Table 3:** Comparison of family process between adolescents with and without health risk behaviors (*n* = 805).

Family process	Non-smokers *n* = 733 Mean (SD)	Smokers *n* = 72 Mean (SD)	*t*-test *t* value (*p* value)	Non-drinkers *n* = 330 Mean (SD)	Drinkers *n* = 475 Mean (SD)	*t*-test *t* value (*p* value)
*Family activities (time spent with parents* *(6 items) ^a^*	11.78(4.24)	9.06(4.30)	5.12 (*p* ≤ 0.001) **	11.78 (4.38)	11.35 (4.26)	1.40 (*p* = 0.162)
*Conflicts with parents* *(5 items) ^b^*	5.77 (3.66)	7.24 (4.33)	−3.19 (*p* = 0.002) *	5.29 (3.57)	6.30 (3.77)	−3.79 (*p* ≤ 0.001) **
*Parenting style* *(6 items) ^c^*	11.44 (2.17)	11.13 (2.05)	1.18 (*p* = 0.238)	11.42 (2.25)	11.42 (2.11)	−0.019 (*p* = 0.985)
*parental support* *(3 items) ^d^*	7.99(2.41)	6.81(2.84)	3.90 (*p* ≤ 0.001) **	8.33(2.47)	7.58 (2.43)	4.26 (*p* ≤ 0.001) **
*parental control* *(3 items) ^d^*	3.45 (2.24)	4.32 (2.54)	−3.10 (*p* = 0.002) *	3.09 (2.38)	3.83 (2.16)	−4.52 (*p* ≤ 0.001) **

^a^ Composite score ranges from 0–24; ^b^ Composite score ranges from 0–20; ^c^ Composite score ranges from 0–24; ^d^ Composite score ranges from 0–12. ** *p* ≤ 0.001; * *p* ≤ 0.05.

### Factors Contributing to Substance Use by Adolescents

Factors that contribute to substance use among the adolescents in this survey were identified using logistic regression. All statistically significantly differences between the users and non-users of substances were included in the analysis. The logistic regression analyses were carried out in two steps for each type of substance use (smoking or drinking). The first set of analyses was conducted by inputting all family-related factors. The second step in the analyses was then done by adding the variables relating to friends.

Table 4 shows that when only family-related factors were analyzed, the factors contributing to adolescent smoking were: having a mother who smokes (OR = 4.633, 95% CI = 1.87–11.49, *p* ≤ 0.001), having a sibling who smokes (OR = 3.16, 95% CI = 1.51–6.64, *p* = 0.0012), and having parents who are “authoritarian” (OR = 1.856, 95% CI = 1.185–2.905, *p* = 0.007). Adolescents were less likely to smoke if they were satisfied with their father’s fulfillment of his role (OR = 0.478, 95% CI = 0.23–0.99, *p* = 0.048), and satisfied with their own self (OR = 0.410, 95% CI = 0.185–0.905, *p* ≤ 0.027). When friends’ factors were added in the second step, only the factors relating to siblings who smoke (OR = 2.61, 95% CI = 1.118–6.09, *p* = 0.027) and satisfaction with oneself (OR = 0.274, 95% CI = 0.121–0.624, *p* = 0.002) remained. Having friends who smoked (OR = 5.446, 95% CI = 2.608–11.374, *p* ≤ 0.001), and friends who invite one to smoke appeared to be the dominant contributors to adolescent smoking with OR = 10.455, 95% CI = 4.434–24.649, *p* ≤ 0.001).

**Table 4:** Logistic regression analysis *^#^* of risk behaviors of adolescents (smoking cigarettes & drinking).

Factors contributing to smoking		Odds ratio	95% CI	*p* value
***Only family factors are considered***				
	Mother smokes	4.633	1.869–11.485	≤0.001 **
	Sibling smokes	3.160	1.505–6.635	0.002 *
	Satisfaction with father	0.478	0.230–0.995	0.048 *
	Satisfaction with self	0.410	0.185–0.905	0.027 *
	Authoritarian parenting	1.856	1.185–2.905	0.007 *
***Friends’ components are added***				
	Sibling smokes	2.610	1.118–6.094	0.027 *
	Satisfaction with self	0.274	0.121–0.624	0.002 *
	Friends smoke	5.446	2.608–11.374	≤0.001 **
	Friend’s invitation to smoke	10.455	4.434–24.649	≤0.001 **
**Factors contributing to drinking**		**Odds ratio**	**95% CI**	***p* value**
***Only family factors are considered***				
	Sibling drinks	4.531	2.174–9.444	≤0.001 **
	Permissive parenting	0.885	0.786–0.996	0.042 *
	Conflicts with parents	1.423	1.004–2.018	0.048 *
**W*hen friends’ components are added***				
	Sibling drinks	3.607	1.510–8.617	0.004 *
	Conflicts with parents	1.529	1.010–2.313	0.045 *
	Friends drink	1.894	1.083–3.311	0.025 *
	Friend’s invitation to drink	11.825	7.715–18.126	≤0.001 **

*^#^ Method = Forward LR*; * *p* ≤ 0.05; ** *p* ≤ 0.001.

When only family-related factors were analyzed, the factors contributing to adolescent drinking were: having a sibling who drinks (OR = 4.53, 95% CI = 2.174–9.444, *p* ≤ 0.001) and having conflicts with one’s parents (OR = 1.423, 95% CI = 1.004–2.018, *p* = 0.048). Those who have parents who are “permissive” were less likely to drink (OR = 0.885, 95% CI = 0.786–0.996, *p* = 0.042). When friends’ factors were added in the second step, both the factors of having siblings who drink (OR = 3.607, 95% CI = 1.51–8.62, *p* = 0.004) and conflicts with parents (OR = 1.529, 95% CI = 1.01–2.31, *p* = 0.045) remained significant. Having friends who drank (OR = 1.894, 95% CI = 1.083–3.311, *p* = 0.025), and having been invited by friends to drink appeared to be the dominant contributors to adolescent drinking with OR = 11.825, 95% CI = 7.715–18.126, *p* ≤ 0.001) (Table 4).

## 4. Discussion

A higher proportion of male students (73.4%) than female students were recruited in this survey due to the participation of one all-boys school, and the gender distribution does not represent the normal distribution in the territory. The prevalence of smoking fathers (44.6%) and mothers (5.6%) in this survey was higher than the prevalence of smoking males (19.9%) and females (3.0%) in Hong Kong [35]. With the participants coming from the Sham Shui Po District of Hong Kong, a district populated by less affluent families, this could reflect the significance association between socio-economic status (SES) and smoking behaviors [36].

The reported prevalence of smoking among the adolescents in this survey (2.7%) is very close to that reported for adolescents in Hong Kong (2.5%) in 2010 [35], indicating that the sample was representative of this population in Hong Kong. This prevalence was relatively low when compared to those found in Western countries, such as 18.1% in the United States [37] and 25% in the United Kingdom [38].

At 56%, the prevalence of those in this study under the age of 18 who had ever tried to drink alcohol is alarming, indicating that the use of alcohol in social activities is finding increasing acceptance among teens. This poses a risk of adolescents progressing to becoming binge drinkers, which could impact on their physical and psychological well-being. Parents and healthcare professionals should be cautioned about the rise of such activities and of the need to be aware of the need to closely monitor the drinking behaviors of youth.

It has long been recognized that family plays an important role in the psychological well-being and health risk behaviors of adolescents [10,39]. A wide range of family factors including family structure, family process, parenting styles, and the smoking habits of family members, have been shown to be associated with adolescent smoking and drinking [40,41]. Although the findings in the present study show that the smoking habits of fathers, mothers, and siblings were all linked to the acquisition by adolescents of these behaviors that are potentially harmful to their health, maternal and sibling smoking had a greater impact on adolescent smoking and drinking than paternal smoking. Both maternal and sibling smoking were identified as predictors of adolescent smoking (OR = 4.63 and 3.16, respectively), a finding consistent with other studies [42]. Our results show that having siblings who smoke is a more influential factor in adolescent smoking than having parents who smoke, as its effect remained significant with regard to both smoking and drinking behaviors even after the inclusion in the analyses of factors related to friends. Similar findings have been observed in other studies [43].

Recent research suggests that family structure is associated with behavior by adolescents that puts their health at risk. The results of this study show that adolescents in single-parent households were more likely to smoke and drink than those who live with both parents. This is possibly due to reduced parental contact, such as the absence of a father [44] or to economic hardship resulting from the need to live on the income of just one parent [45] inducing stress in the adolescents. However, this effect was moderated when other family processes such as parental support and control, and conflict with parents, were taken into account. This suggests that other factors may have a greater bearing on behavior by adolescents that is harmful to their health, consistent with the findings of other studies [40,46].

The parenting process in terms of parent-child relations and parenting styles is closely related to adolescent psychological well-being and problem behavior. The results of this study show that those adolescents who spend more time engaged in activities with their parents are at a lower risk of behaving in ways that are harmful to their health, while a poor parent-child relationship as indicated by frequent parent-child conflicts puts adolescents at a greater risk of engaging in substance abuse. These findings are supported by other studies [10,11,41].

Parenting style is an important factor influencing the psychosocial development of adolescents, their use of substances, and delinquency [47]. The results of this study are consistent with those of previous studies that found that parental support and control affect the uptake of behavior by adolescents that puts their health at risk [41,48,49]. A nationwide study in the United States has found that both too much and too little parental control contribute to adolescent delinquency [49]. While it was thought that authoritarian parenting impacts on adolescents regardless of ethnic background, a study among Latino found that increases in parental control, because of their tradition and culture norm, function positively for Latino families and protects adolescents against problem behaviors [50]. This study among Chinese, found that authoritarian (low support and high control) and permissive (high support and low control) parenting were contributors to drinking and smoking by adolescents. Parents who are nurturing and supportive could enhance feelings of self-esteem and security in their children; however, without appropriate monitoring or control, supportive parenting could also be ineffective. An appropriate level of support and control on the part of parents could improve parent-child relations, causing parents to be more successful in guiding adolescents to engage in positive health behavior.

A large number of studies have documented that peers play an important role in the development of adolescent risk behavior. Our findings support the notion that the influence of friends is highly predictive of whether or not an adolescent will engage in smoking and drinking. These results are supported by previous studies [21,39]. In this study, non-smoking adolescents had friends who objected to smoking, suggesting that the influence of friends could either promote or deter risky behavior, as has been found in other studies [22,33]. The pressure to gain acceptance among friends by smoking is common among adolescents. Understanding the influence of peers could enable practitioners to design appropriate measures to prevent the development of risky behavior among youth.

Substance use could compromise one’s health. Although the cumulative negative effects of smoking and drinking are slow, and signs and symptoms may not be apparent in adolescents, early intervention is desirable to prevent engagement in risky behaviors.

### Limitations of the Study

There are several limitations to this study. First, the study’s population was not representative of the gender distribution in Hong Kong. Participants were drawn from a less affluent district with families of low socio-economic status, making the findings not generalizable. Second, the cross-sectional nature of the study limits our ability to make causal inferences on the assessed variables. Third, the analyses that were conducted were based on self-reported data, making response bias a possibility.

## 5. Conclusions

Using tobacco and drinking alcohol are leading causes of many preventable diseases. Family processes and the influence of friends will influence engagement in such behavior during adolescence, making adolescents susceptible to health risks later in life.

Familial influences are seen as important factors in the development of adolescents. Children look up to their parents as role models; therefore, smoking on the part of parents and siblings will be regarded by children as acceptable behavior, which they are likely to emulate. The ready availability of cigarettes at home also encourages teenagers to try smoking, which could lead to the development of this risky behavior. Parents and siblings, being the first persons that children interact with, exert immense influence on children’s behaviors by acting as role models. Recognizing the negative role that they may play on the development of behavior among their children or younger siblings that could be harmful to their health, family members who smoke should be encouraged to quit.

Besides serving as role models, parents also provide support and control to guide their children in the course of their development. The quality of the parent-child relationship is another factor influencing the development of risky behavior. A poor parent-child relationship, as reflected by less time spent in activities together and increased conflict with parents, is a factor contributing to engagement in risky behavior. Parenting with warmth, love, care, acceptance, respect, reason, and the appropriate level of monitoring will encourage positive psychosocial development in children. Neither an authoritarian (low support but high control) nor a permissive (high support but low control) parenting style is seen as ideal, as shown in this study. High support without an appropriate level of control or vice versa could contribute to engagement in risky behavior by adolescents, such as smoking and drinking.

As adolescents begin to socialize with friends, the role of peers becomes important. Friends of smokers are far more likely to transition to tobacco use/drinking than friends of non-smokers/non-drinkers. In this study, the smoking and drinking habits of friends were important predictors of the uptake by adolescents of such behaviors. Having a friend who smokes will increase the odds that an adolescent will smoke by 5.45 times, rising to 10.46 times when the adolescent is invited by a friend to smoke. Having a friend who drinks will increase the odds that an adolescent will drink by 1.89 times, increasing to 11.825 times when invited by a friend to drink.

In this study, the smoking status of family members, parent-child conflicts, parenting styles, and the influence of friends were all found to correlate with the development of smoking and drinking among adolescents. This information is valuable for planning programs for the prevention and cessation of risky behavior among adolescents.

### Community-Based Family-Centered Interventions

Families should be supported in a respectful approach that views the family as central to the well-being of adolescents. Any intervention needs to recognize the strength of the parent-child relationship and to build on this strength to establish warmth and support in families. It needs to provide a range of activities designed to strengthen family functioning by improving interpersonal relations, parenting, and monitoring skills. Family-centered interventions should also include counseling, communication skills, and information on enhancing parenting abilities; advocating for families; and connecting families with the support services that they need to achieve a nurturing and stable family environment.

Healthy families also facilitate communication between parents and children and have a good influence on an adolescent’s selection of friends, both of which will greatly influence an adolescent’s choice of health behaviors. In order to reduce the incidence and prevalence of behaviors risky to health among adolescents, communities should make an effort to enhance those factors that are protective of families with adolescents. The result will be beneficial for the health of adolescents, families, and the community.
